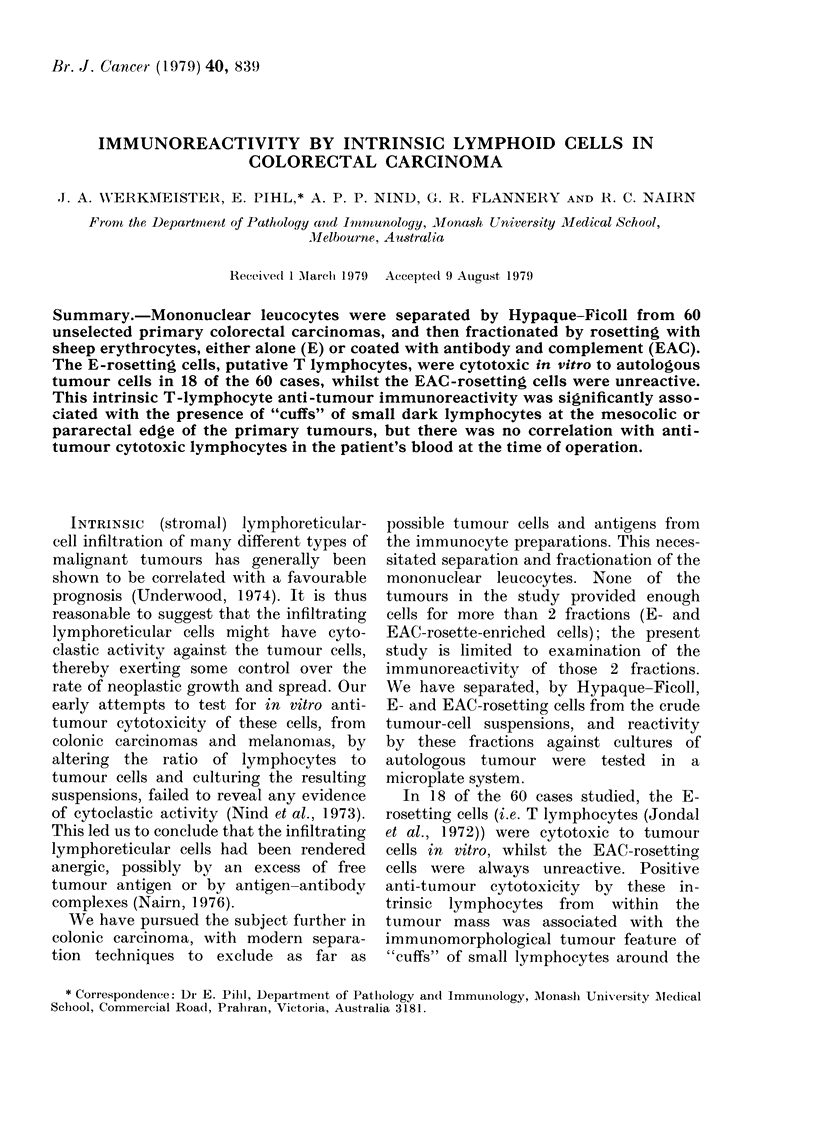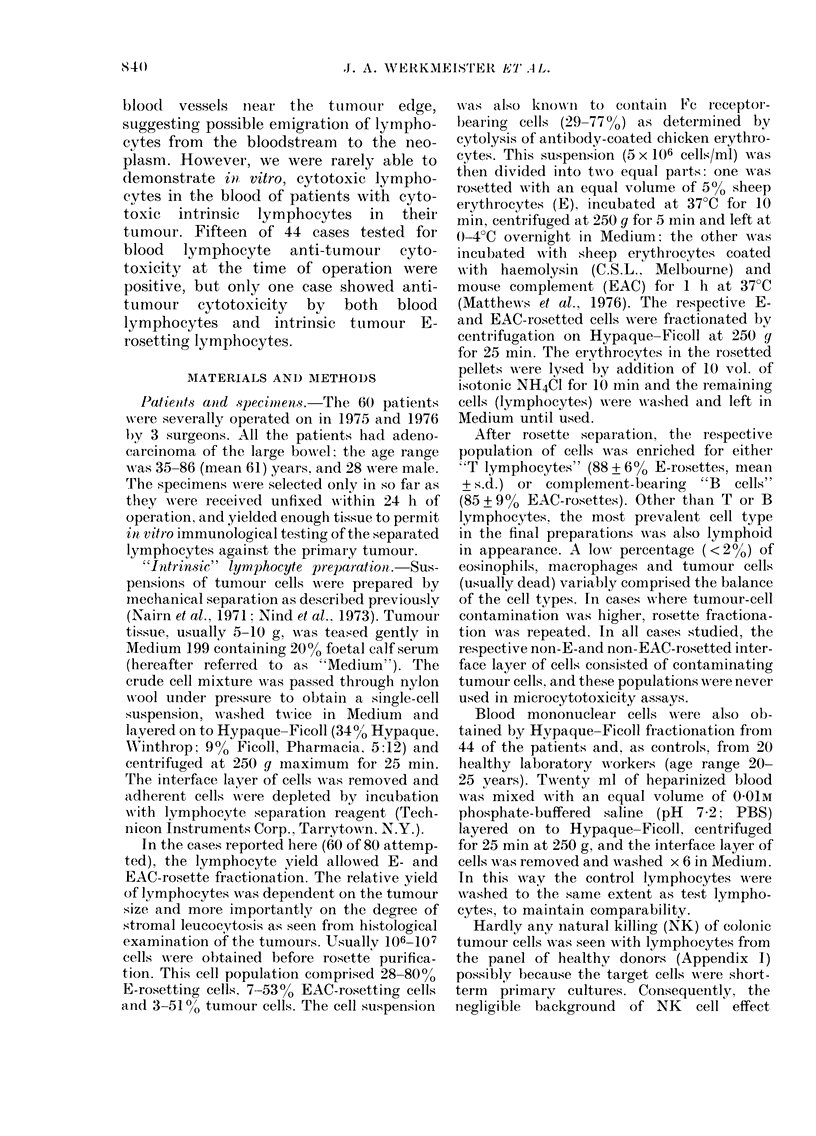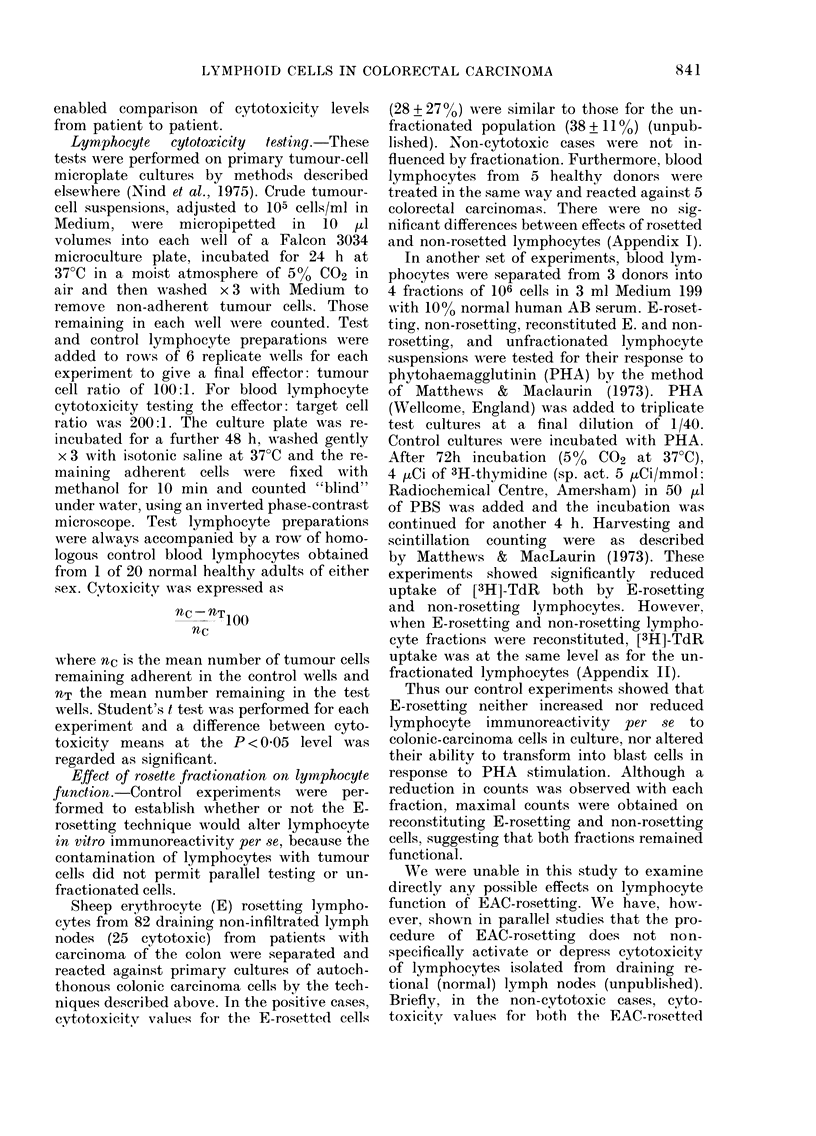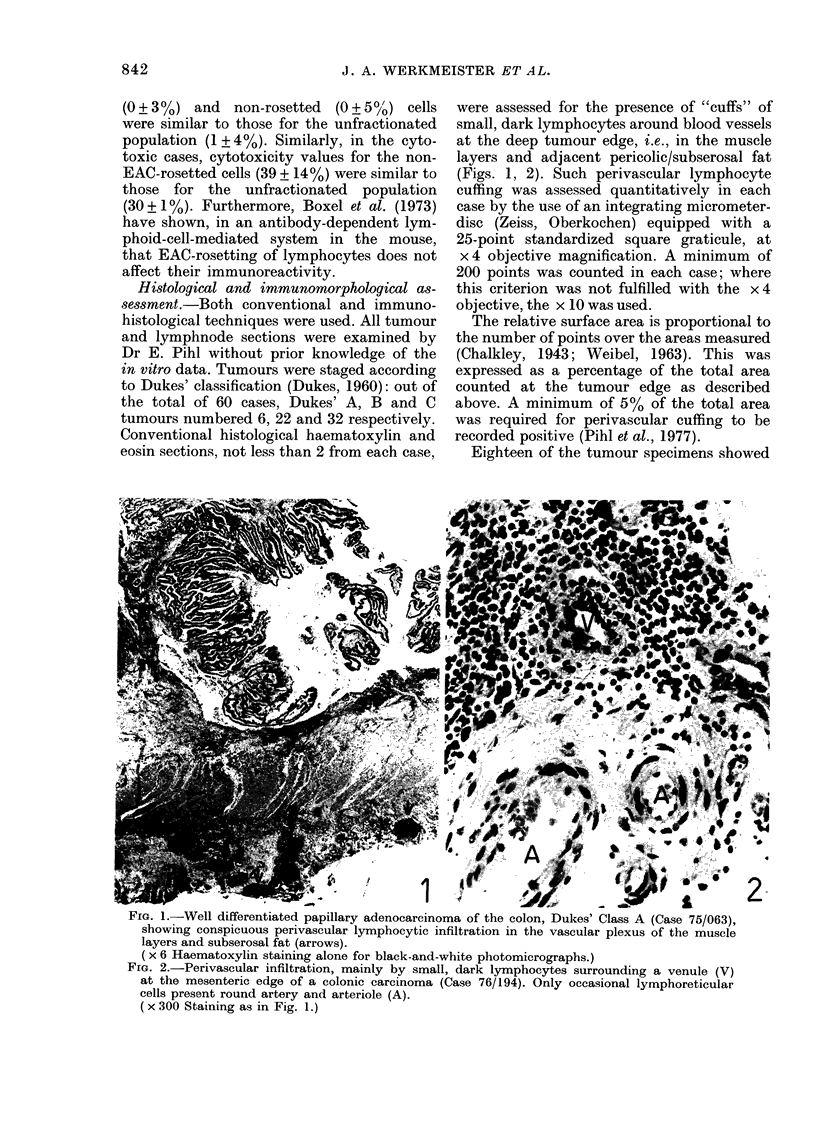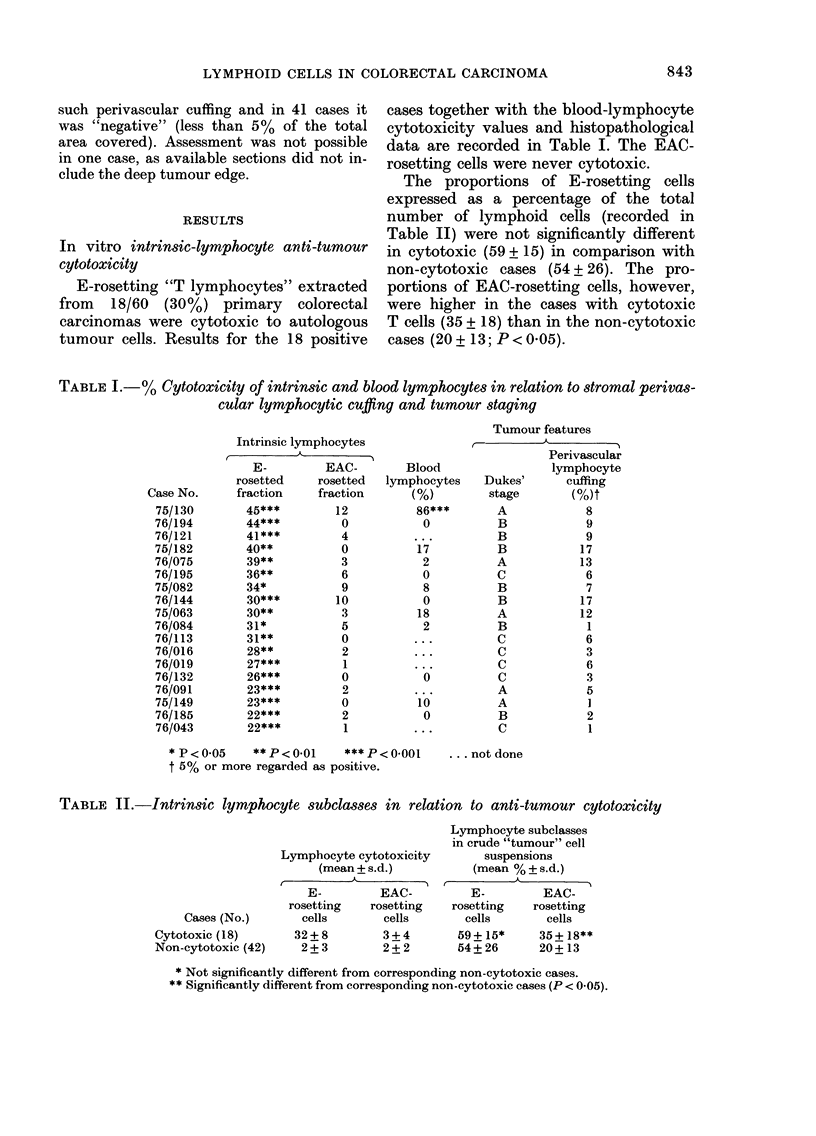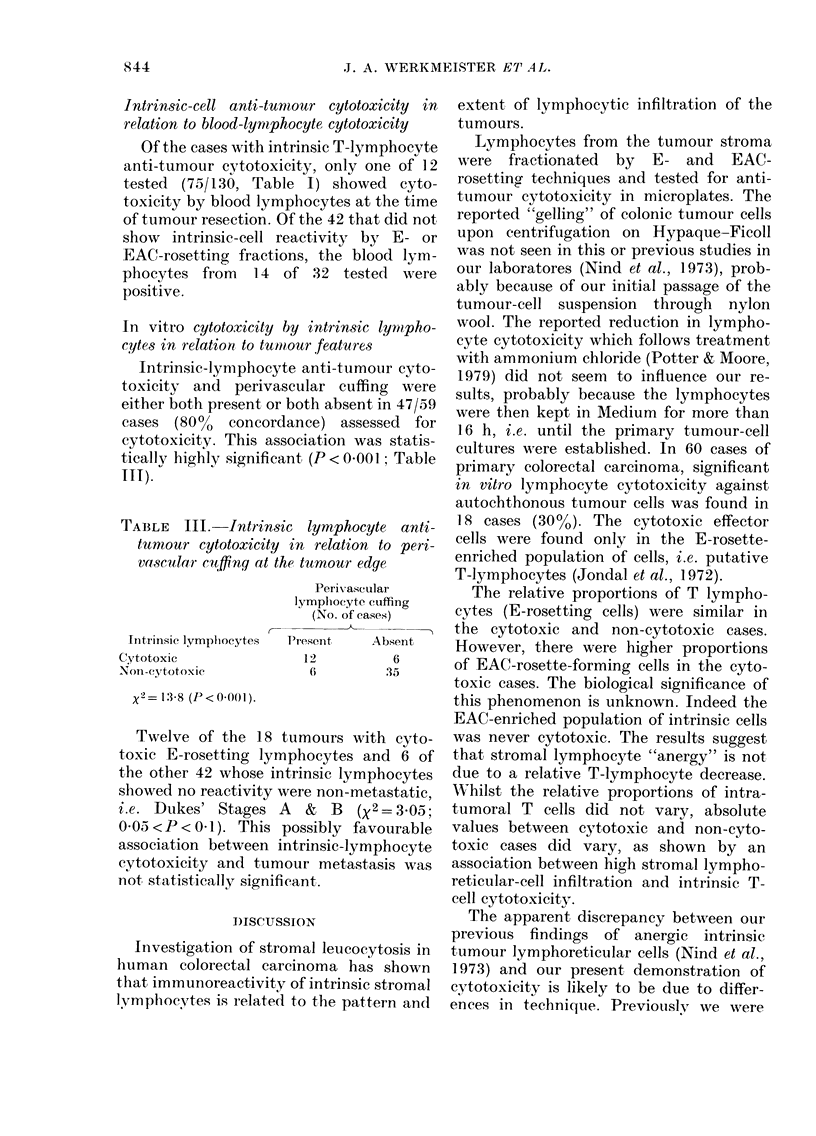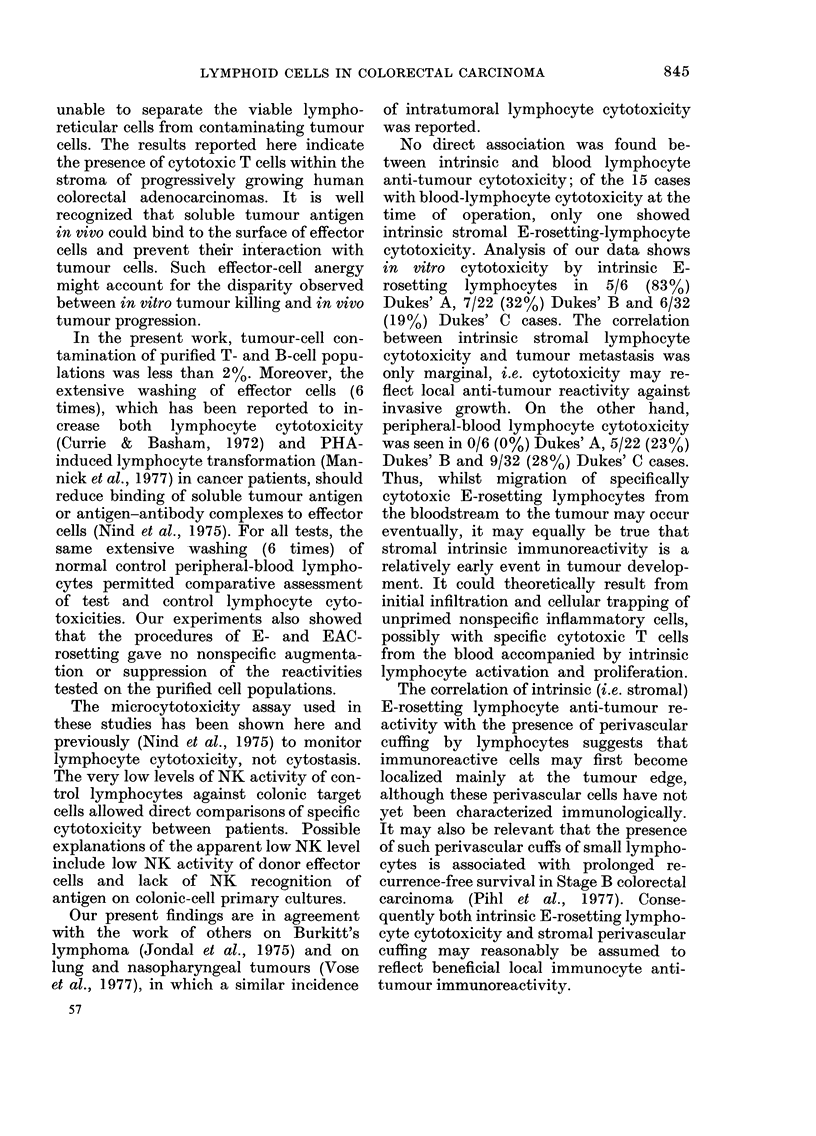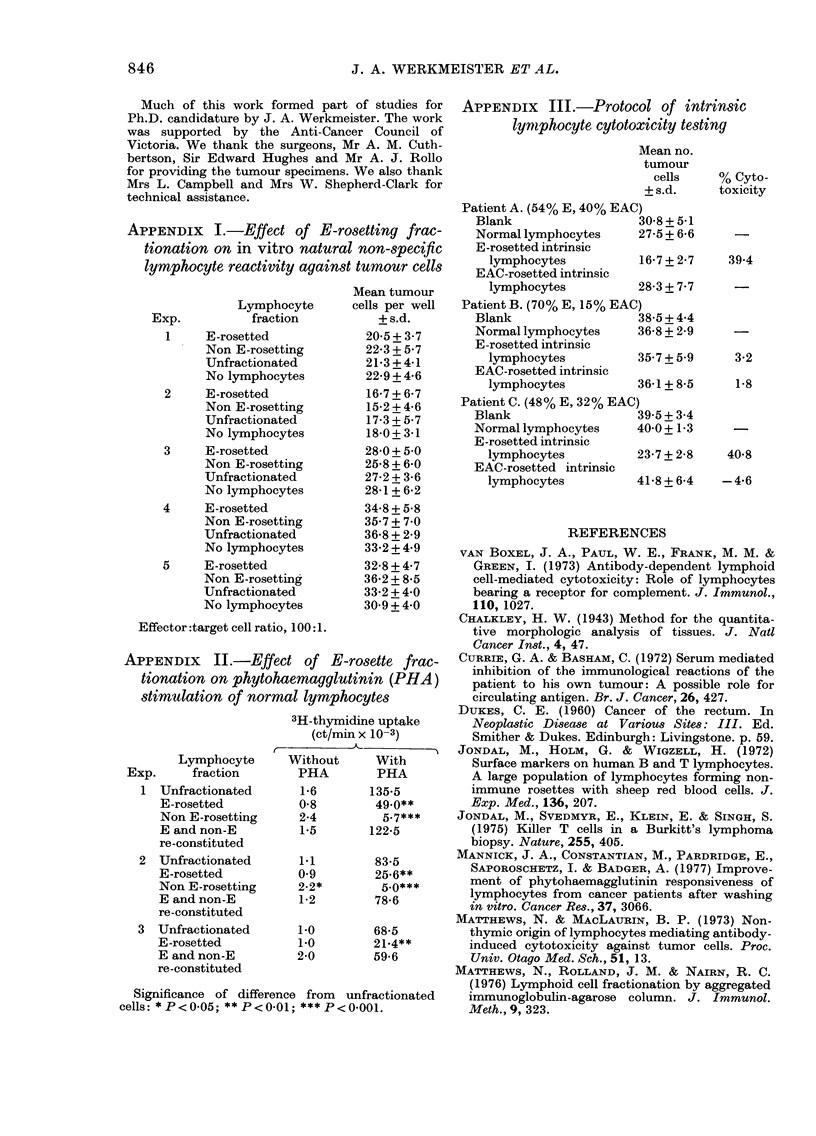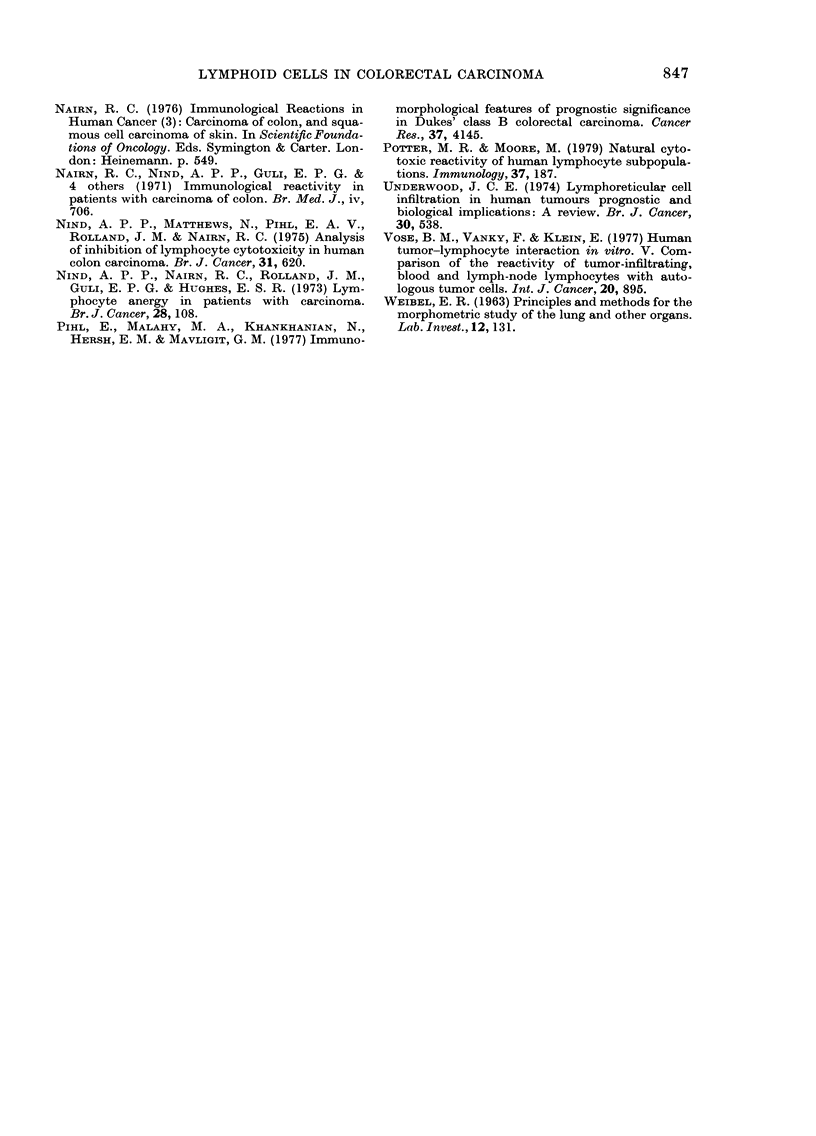# Immunoreactivity by intrinsic lymphoid cells in colorectal carcinoma.

**DOI:** 10.1038/bjc.1979.274

**Published:** 1979-12

**Authors:** J. A. Werkmeister, E. Pihl, A. P. Nind, G. R. Flannery, R. C. Nairn

## Abstract

**Images:**


*Br. J. Cancer* (1979) **40**, 839

### IMMUNOREACTIVITY BY INTRINSIC LYMPHOID CELLS IN COLORECTAL CARCINOMA

J. A. WERKMEISTER, E. PIHL,* A. P. P. NIND, G. R. FLANNERY AND R. C. NAIRN

*From the Department of Pathology and Immunology, Monash University Medical School, Melbourne, Australia*

Received 1 March 1979 Accepted 9 August 1979

**Summary.—Mononuclear leucocytes were separated by Hypaque–Ficoll from 60 unselected primary colorectal carcinomas, and then fractionated by rosetting with sheep erythrocytes, either alone (E) or coated with antibody and complement (EAC). The E-rosetting cells, putative T lymphocytes, were cytotoxic *in vitro* to autologous tumour cells in 18 of the 60 cases, whilst the EAC-rosetting cells were unreactive. This intrinsic T-lymphocyte anti-tumour immunoreactivity was significantly associated with the presence of "cuffs" of small dark lymphocytes at the mesocolic or pararectal edge of the primary tumours, but there was no correlation with anti-tumour cytotoxic lymphocytes in the patient's blood at the time of operation.**

INTRINSIC (stromal) lymphoreticular-cell infiltration of many different types of malignant tumours has generally been shown to be correlated with a favourable prognosis (Underwood, 1974). It is thus reasonable to suggest that the infiltrating lymphoreticular cells might have cytoclastic activity against the tumour cells, thereby exerting some control over the rate of neoplastic growth and spread. Our early attempts to test for *in vitro* anti-tumour cytotoxicity of these cells, from colonic carcinomas and melanomas, by altering the ratio of lymphocytes to tumour cells and culturing the resulting suspensions, failed to reveal any evidence of cytoclastic activity (Nind *et al.*, 1973). This led us to conclude that the infiltrating lymphoreticular cells had been rendered anergic, possibly by an excess of free tumour antigen or by antigen–antibody complexes (Nairn, 1976).

We have pursued the subject further in colonic carcinoma, with modern separation techniques to exclude as far as possible tumour cells and antigens from the immunocyte preparations. This necessitated separation and fractionation of the mononuclear leucocytes. None of the tumours in the study provided enough cells for more than 2 fractions (E- and EAC-rosette-enriched cells); the present study is limited to examination of the immunoreactivity of those 2 fractions. We have separated, by Hypaque–Ficoll, E- and EAC-rosetting cells from the crude tumour-cell suspensions, and reactivity by these fractions against cultures of autologous tumour were tested in a microplate system.

In 18 of the 60 cases studied, the E-rosetting cells (*i.e.* T lymphocytes (Jondal *et al.*, 1972)) were cytotoxic to tumour cells *in vitro*, whilst the EAC-rosetting cells were always unreactive. Positive anti-tumour cytotoxicity by these intrinsic lymphocytes from within the tumour mass was associated with the immunomorphological tumour feature of "cuffs" of small lymphocytes around the

* Correspondence: Dr E. Pihl, Department of Pathology and Immunology, Monash University Medical School, Commercial Road, Prahran, Victoria, Australia 3181.

blood vessels near the tumour edge, suggesting possible emigration of lymphocytes from the bloodstream to the neoplasm. However, we were rarely able to demonstrate *in vitro*, cytotoxic lymphocytes in the blood of patients with cytotoxic intrinsic lymphocytes in their tumour. Fifteen of 44 cases tested for blood lymphocyte anti-tumour cytotoxicity at the time of operation were positive, but only one case showed anti-tumour cytotoxicity by both blood lymphocytes and intrinsic tumour E-rosetting lymphocytes.

### MATERIALS AND METHODS

*Patients and specimens.*—The 60 patients were severally operated on in 1975 and 1976 by 3 surgeons. All the patients had adenocarcinoma of the large bowel; the age range was 35–86 (mean 61) years, and 28 were male. The specimens were selected only in so far as they were received unfixed within 24 h of operation, and yielded enough tissue to permit *in vitro* immunological testing of the separated lymphocytes against the primary tumour.

*"Intrinsic" lymphocyte preparation.*—Suspensions of tumour cells were prepared by mechanical separation as described previously (Nairn *et al.*, 1971; Nind *et al.*, 1973). Tumour tissue, usually 5–10 g, was teased gently in Medium 199 containing 20% foetal calf serum (hereafter referred to as "Medium"). The crude cell mixture was passed through nylon wool under pressure to obtain a single-cell suspension, washed twice in Medium and layered on to Hypaque–Ficoll (34% Hypaque, Winthrop; 9% Ficoll, Pharmacia, 5:12) and centrifuged at 250 *g* maximum for 25 min. The interface layer of cells was removed and adherent cells were depleted by incubation with lymphocyte separation reagent (Technicon Instruments Corp., Tarrytown, N.Y.).

In the cases reported here (60 of 80 attempted), the lymphocyte yield allowed E- and EAC-rosette fractionation. The relative yield of lymphocytes was dependent on the tumour size and more importantly on the degree of stromal leucocytosis as seen from histological examination of the tumours. Usually $10^6$–$10^7$ cells were obtained before rosette purification. This cell population comprised 28–80% E-rosetting cells, 7–53% EAC-rosetting cells and 3–51% tumour cells. The cell suspension was also known to contain Fc receptor-bearing cells (29–77%) as determined by cytolysis of antibody-coated chicken erythrocytes. This suspension ($5 \times 10^6$ cells/ml) was then divided into two equal parts: one was rosetted with an equal volume of 5% sheep erythrocytes (E), incubated at 37°C for 10 min, centrifuged at 250 *g* for 5 min and left at 0–4°C overnight in Medium; the other was incubated with sheep erythrocytes coated with haemolysin (C.S.L., Melbourne) and mouse complement (EAC) for 1 h at 37°C (Matthews *et al.*, 1976). The respective E- and EAC-rosetted cells were fractionated by centrifugation on Hypaque–Ficoll at 250 *g* for 25 min. The erythrocytes in the rosetted pellets were lysed by addition of 10 vol. of isotonic $NH_4Cl$ for 10 min and the remaining cells (lymphocytes) were washed and left in Medium until used.

After rosette separation, the respective population of cells was enriched for either "T lymphocytes" ($88 \pm 6\%$ E-rosettes, mean $\pm$s.d.) or complement-bearing "B cells" ($85 \pm 9\%$ EAC-rosettes). Other than T or B lymphocytes, the most prevalent cell type in the final preparations was also lymphoid in appearance. A low percentage ($<2\%$) of eosinophils, macrophages and tumour cells (usually dead) variably comprised the balance of the cell types. In cases where tumour-cell contamination was higher, rosette fractionation was repeated. In all cases studied, the respective non-E-and non-EAC-rosetted interface layer of cells consisted of contaminating tumour cells, and these populations were never used in microcytotoxicity assays.

Blood mononuclear cells were also obtained by Hypaque–Ficoll fractionation from 44 of the patients and, as controls, from 20 healthy laboratory workers (age range 20–25 years). Twenty ml of heparinized blood was mixed with an equal volume of 0·01M phosphate-buffered saline (pH 7·2; PBS) layered on to Hypaque–Ficoll, centrifuged for 25 min at 250 g, and the interface layer of cells was removed and washed $\times 6$ in Medium. In this way the control lymphocytes were washed to the same extent as test lymphocytes, to maintain comparability.

Hardly any natural killing (NK) of colonic tumour cells was seen with lymphocytes from the panel of healthy donors (Appendix I) possibly because the target cells were short-term primary cultures. Consequently, the negligible background of NK cell effect enabled comparison of cytotoxicity levels from patient to patient.

*Lymphocyte cytotoxicity testing.*—These tests were performed on primary tumour-cell microplate cultures by methods described elsewhere (Nind *et al.*, 1975). Crude tumour-cell suspensions, adjusted to $10^5$ cells/ml in Medium, were micropipetted in 10 $\mu$l volumes into each well of a Falcon 3034 microculture plate, incubated for 24 h at 37°C in a moist atmosphere of 5% $CO_2$ in air and then washed ×3 with Medium to remove non-adherent tumour cells. Those remaining in each well were counted. Test and control lymphocyte preparations were added to rows of 6 replicate wells for each experiment to give a final effector: tumour cell ratio of 100:1. For blood lymphocyte cytotoxicity testing the effector: target cell ratio was 200:1. The culture plate was re-incubated for a further 48 h, washed gently ×3 with isotonic saline at 37°C and the remaining adherent cells were fixed with methanol for 10 min and counted "blind" under water, using an inverted phase-contrast microscope. Test lymphocyte preparations were always accompanied by a row of homologous control blood lymphocytes obtained from 1 of 20 normal healthy adults of either sex. Cytoxicity was expressed as

$$\frac{n_C - n_T}{n_C}100$$

where $n_C$ is the mean number of tumour cells remaining adherent in the control wells and $n_T$ the mean number remaining in the test wells. Student's $t$ test was performed for each experiment and a difference between cytotoxicity means at the $P < 0{\cdot}05$ level was regarded as significant.

*Effect of rosette fractionation on lymphocyte function.*—Control experiments were performed to establish whether or not the E-rosetting technique would alter lymphocyte *in vitro* immunoreactivity *per se*, because the contamination of lymphocytes with tumour cells did not permit parallel testing or unfractionated cells.

Sheep erythrocyte (E) rosetting lymphocytes from 82 draining non-infiltrated lymph nodes (25 cytotoxic) from patients with carcinoma of the colon were separated and reacted against primary cultures of autochthonous colonic carcinoma cells by the techniques described above. In the positive cases, cytotoxicity values for the E-rosetted cells ($28 \pm 27\%$) were similar to those for the unfractionated population ($38 \pm 11\%$) (unpublished). Non-cytotoxic cases were not influenced by fractionation. Furthermore, blood lymphocytes from 5 healthy donors were treated in the same way and reacted against 5 colorectal carcinomas. There were no significant differences between effects of rosetted and non-rosetted lymphocytes (Appendix I).

In another set of experiments, blood lymphocytes were separated from 3 donors into 4 fractions of $10^6$ cells in 3 ml Medium 199 with 10% normal human AB serum. E-rosetting, non-rosetting, reconstituted E. and non-rosetting, and unfractionated lymphocyte suspensions were tested for their response to phytohaemagglutinin (PHA) by the method of Matthews & Maclaurin (1973). PHA (Wellcome, England) was added to triplicate test cultures at a final dilution of 1/40. Control cultures were incubated with PHA. After 72h incubation (5% $CO_2$ at 37°C), 4 $\mu$Ci of $^3$H-thymidine (sp. act. 5 $\mu$Ci/mmol; Radiochemical Centre, Amersham) in 50 $\mu$l of PBS was added and the incubation was continued for another 4 h. Harvesting and scintillation counting were as described by Matthews & MacLaurin (1973). These experiments showed significantly reduced uptake of [$^3$H]-TdR both by E-rosetting and non-rosetting lymphocytes. However, when E-rosetting and non-rosetting lymphocyte fractions were reconstituted, [$^3$H]-TdR uptake was at the same level as for the unfractionated lymphocytes (Appendix II).

Thus our control experiments showed that E-rosetting neither increased nor reduced lymphocyte immunoreactivity *per se* to colonic-carcinoma cells in culture, nor altered their ability to transform into blast cells in response to PHA stimulation. Although a reduction in counts was observed with each fraction, maximal counts were obtained on reconstituting E-rosetting and non-rosetting cells, suggesting that both fractions remained functional.

We were unable in this study to examine directly any possible effects on lymphocyte function of EAC-rosetting. We have, however, shown in parallel studies that the procedure of EAC-rosetting does not non-specifically activate or depress cytotoxicity of lymphocytes isolated from draining retional (normal) lymph nodes (unpublished). Briefly, in the non-cytotoxic cases, cytotoxicity values for both the EAC-rosetted

(0 ± 3%) and non-rosetted (0 ± 5%) cells were similar to those for the unfractionated population (1 ± 4%). Similarly, in the cytotoxic cases, cytotoxicity values for the non-EAC-rosetted cells (39 ± 14%) were similar to those for the unfractionated population (30 ± 1%). Furthermore, Boxel *et al.* (1973) have shown, in an antibody-dependent lymphoid-cell-mediated system in the mouse, that EAC-rosetting of lymphocytes does not affect their immunoreactivity.

*Histological and immunomorphological assessment.*—Both conventional and immunohistological techniques were used. All tumour and lymphnode sections were examined by Dr E. Pihl without prior knowledge of the *in vitro* data. Tumours were staged according to Dukes' classification (Dukes, 1960): out of the total of 60 cases, Dukes' A, B and C tumours numbered 6, 22 and 32 respectively. Conventional histological haematoxylin and eosin sections, not less than 2 from each case, were assessed for the presence of "cuffs" of small, dark lymphocytes around blood vessels at the deep tumour edge, *i.e.*, in the muscle layers and adjacent pericolic/subserosal fat (Figs. 1, 2). Such perivascular lymphocyte cuffing was assessed quantitatively in each case by the use of an integrating micrometer-disc (Zeiss, Oberkochen) equipped with a 25-point standardized square graticule, at × 4 objective magnification. A minimum of 200 points was counted in each case; where this criterion was not fulfilled with the × 4 objective, the × 10 was used.

The relative surface area is proportional to the number of points over the areas measured (Chalkley, 1943; Weibel, 1963). This was expressed as a percentage of the total area counted at the tumour edge as described above. A minimum of 5% of the total area was required for perivascular cuffing to be recorded positive (Pihl *et al.*, 1977).

Eighteen of the tumour specimens showed

FIG. 1.—Well differentiated papillary adenocarcinoma of the colon, Dukes' Class A (Case 75/063), showing conspicuous perivascular lymphocytic infiltration in the vascular plexus of the muscle layers and subserosal fat (arrows).
( × 6 Haematoxylin staining alone for black-and-white photomicrographs.)

FIG. 2.—Perivascular infiltration, mainly by small, dark lymphocytes surrounding a venule (V) at the mesenteric edge of a colonic carcinoma (Case 76/194). Only occasional lymphoreticular cells present round artery and arteriole (A).
( × 300 Staining as in Fig. 1.)

such perivascular cuffing and in 41 cases it was "negative" (less than 5% of the total area covered). Assessment was not possible in one case, as available sections did not include the deep tumour edge.

### RESULTS

### In vitro *intrinsic-lymphocyte anti-tumour cytotoxicity*

E-rosetting "T lymphocytes" extracted from 18/60 (30%) primary colorectal carcinomas were cytotoxic to autologous tumour cells. Results for the 18 positive cases together with the blood-lymphocyte cytotoxicity values and histopathological data are recorded in Table I. The EAC-rosetting cells were never cytotoxic.

The proportions of E-rosetting cells expressed as a percentage of the total number of lymphoid cells (recorded in Table II) were not significantly different in cytotoxic (59 ± 15) in comparison with non-cytotoxic cases (54 ± 26). The proportions of EAC-rosetting cells, however, were higher in the cases with cytotoxic T cells (35 ± 18) than in the non-cytotoxic cases (20 ± 13; $P < 0.05$).

TABLE I.—% *Cytotoxicity of intrinsic and blood lymphocytes in relation to stromal perivascular lymphocytic cuffing and tumour staging*

| | Intrinsic lymphocytes | | | Tumour features | |
|---|---|---|---|---|---|
| Case No. | E-rosetted fraction | EAC-rosetted fraction | Blood lymphocytes (%) | Dukes' stage | Perivascular lymphocyte cuffing (%)† |
| 75/130 | 45*** | 12 | 86*** | A | 8 |
| 76/194 | 44*** | 0 | 0 | B | 9 |
| 76/121 | 41*** | 4 | ... | B | 9 |
| 75/182 | 40** | 0 | 17 | B | 17 |
| 76/075 | 39** | 3 | 2 | A | 13 |
| 76/195 | 36** | 6 | 0 | C | 6 |
| 75/082 | 34* | 9 | 8 | B | 7 |
| 76/144 | 30*** | 10 | 0 | B | 17 |
| 75/063 | 30** | 3 | 18 | A | 12 |
| 76/084 | 31* | 5 | 2 | B | 1 |
| 76/113 | 31** | 0 | ... | C | 6 |
| 76/016 | 28** | 2 | ... | C | 3 |
| 76/019 | 27*** | 1 | ... | C | 6 |
| 76/132 | 26*** | 0 | 0 | C | 3 |
| 76/091 | 23*** | 2 | ... | A | 5 |
| 75/149 | 23*** | 0 | 10 | A | 1 |
| 76/185 | 22*** | 2 | 0 | B | 2 |
| 76/043 | 22*** | 1 | ... | C | 1 |

* $P < 0.05$ ** $P < 0.01$ *** $P < 0.001$ ... not done

† 5% or more regarded as positive.

TABLE II.—*Intrinsic lymphocyte subclasses in relation to anti-tumour cytotoxicity*

| | Lymphocyte cytotoxicity (mean ± s.d.) | | Lymphocyte subclasses in crude "tumour" cell suspensions (mean % ± s.d.) | |
|---|---|---|---|---|
| Cases (No.) | E-rosetting cells | EAC-rosetting cells | E-rosetting cells | EAC-rosetting cells |
| Cytotoxic (18) | 32 ± 8 | 3 ± 4 | 59 ± 15* | 35 ± 18** |
| Non-cytotoxic (42) | 2 ± 3 | 2 ± 2 | 54 ± 26 | 20 ± 13 |

* Not significantly different from corresponding non-cytotoxic cases.
** Significantly different from corresponding non-cytotoxic cases ($P < 0.05$).

*Intrinsic-cell anti-tumour cytotoxicity in relation to blood-lymphocyte cytotoxicity*

Of the cases with intrinsic T-lymphocyte anti-tumour cytotoxicity, only one of 12 tested (75/130, Table I) showed cytotoxicity by blood lymphocytes at the time of tumour resection. Of the 42 that did not show intrinsic-cell reactivity by E- or EAC-rosetting fractions, the blood lymphocytes from 14 of 32 tested were positive.

In vitro *cytotoxicity by intrinsic lymphocytes in relation to tumour features*

Intrinsic-lymphocyte anti-tumour cytotoxicity and perivascular cuffing were either both present or both absent in 47/59 cases (80% concordance) assessed for cytotoxicity. This association was statistically highly significant ($P < 0{\cdot}001$; Table III).

TABLE III.—*Intrinsic lymphocyte anti-tumour cytotoxicity in relation to perivascular cuffing at the tumour edge*

| | Perivascular lymphocyte cuffing (No. of cases) | |
|---|---|---|
| Intrinsic lymphocytes | Present | Absent |
| Cytotoxic | 12 | 6 |
| Non-cytotoxic | 6 | 35 |

$\chi^2 = 13{\cdot}8$ ($P < 0{\cdot}001$).

Twelve of the 18 tumours with cytotoxic E-rosetting lymphocytes and 6 of the other 42 whose intrinsic lymphocytes showed no reactivity were non-metastatic, *i.e.* Dukes' Stages A & B ($\chi^2 = 3{\cdot}05$; $0{\cdot}05 < P < 0{\cdot}1$). This possibly favourable association between intrinsic-lymphocyte cytotoxicity and tumour metastasis was not statistically significant.

### DISCUSSION

Investigation of stromal leucocytosis in human colorectal carcinoma has shown that immunoreactivity of intrinsic stromal lymphocytes is related to the pattern and extent of lymphocytic infiltration of the tumours.

Lymphocytes from the tumour stroma were fractionated by E- and EAC-rosetting techniques and tested for anti-tumour cytotoxicity in microplates. The reported "gelling" of colonic tumour cells upon centrifugation on Hypaque–Ficoll was not seen in this or previous studies in our laboratores (Nind *et al.*, 1973), probably because of our initial passage of the tumour-cell suspension through nylon wool. The reported reduction in lymphocyte cytotoxicity which follows treatment with ammonium chloride (Potter & Moore, 1979) did not seem to influence our results, probably because the lymphocytes were then kept in Medium for more than 16 h, *i.e.* until the primary tumour-cell cultures were established. In 60 cases of primary colorectal carcinoma, significant *in vitro* lymphocyte cytotoxicity against autochthonous tumour cells was found in 18 cases (30%). The cytotoxic effector cells were found only in the E-rosette-enriched population of cells, *i.e.* putative T-lymphocytes (Jondal *et al.*, 1972).

The relative proportions of T lymphocytes (E-rosetting cells) were similar in the cytotoxic and non-cytotoxic cases. However, there were higher proportions of EAC-rosette-forming cells in the cytotoxic cases. The biological significance of this phenomenon is unknown. Indeed the EAC-enriched population of intrinsic cells was never cytotoxic. The results suggest that stromal lymphocyte "anergy" is not due to a relative T-lymphocyte decrease. Whilst the relative proportions of intratumoral T cells did not vary, absolute values between cytotoxic and non-cytotoxic cases did vary, as shown by an association between high stromal lymphoreticular-cell infiltration and intrinsic T-cell cytotoxicity.

The apparent discrepancy between our previous findings of anergic intrinsic tumour lymphoreticular cells (Nind *et al.*, 1973) and our present demonstration of cytotoxicity is likely to be due to differences in technique. Previously we were unable to separate the viable lymphoreticular cells from contaminating tumour cells. The results reported here indicate the presence of cytotoxic T cells within the stroma of progressively growing human colorectal adenocarcinomas. It is well recognized that soluble tumour antigen *in vivo* could bind to the surface of effector cells and prevent their interaction with tumour cells. Such effector-cell anergy might account for the disparity observed between *in vitro* tumour killing and *in vivo* tumour progression.

In the present work, tumour-cell contamination of purified T- and B-cell populations was less than 2%. Moreover, the extensive washing of effector cells (6 times), which has been reported to increase both lymphocyte cytotoxicity (Currie & Basham, 1972) and PHA-induced lymphocyte transformation (Mannick *et al.*, 1977) in cancer patients, should reduce binding of soluble tumour antigen or antigen–antibody complexes to effector cells (Nind *et al.*, 1975). For all tests, the same extensive washing (6 times) of normal control peripheral-blood lymphocytes permitted comparative assessment of test and control lymphocyte cytotoxicities. Our experiments also showed that the procedures of E- and EAC-rosetting gave no nonspecific augmentation or suppression of the reactivities tested on the purified cell populations.

The microcytotoxicity assay used in these studies has been shown here and previously (Nind *et al.*, 1975) to monitor lymphocyte cytotoxicity, not cytostasis. The very low levels of NK activity of control lymphocytes against colonic target cells allowed direct comparisons of specific cytotoxicity between patients. Possible explanations of the apparent low NK level include low NK activity of donor effector cells and lack of NK recognition of antigen on colonic-cell primary cultures.

Our present findings are in agreement with the work of others on Burkitt's lymphoma (Jondal *et al.*, 1975) and on lung and nasopharyngeal tumours (Vose *et al.*, 1977), in which a similar incidence of intratumoral lymphocyte cytotoxicity was reported.

No direct association was found between intrinsic and blood lymphocyte anti-tumour cytotoxicity; of the 15 cases with blood-lymphocyte cytotoxicity at the time of operation, only one showed intrinsic stromal E-rosetting-lymphocyte cytotoxicity. Analysis of our data shows *in vitro* cytotoxicity by intrinsic E-rosetting lymphocytes in 5/6 (83%) Dukes' A, 7/22 (32%) Dukes' B and 6/32 (19%) Dukes' C cases. The correlation between intrinsic stromal lymphocyte cytotoxicity and tumour metastasis was only marginal, *i.e.* cytotoxicity may reflect local anti-tumour reactivity against invasive growth. On the other hand, peripheral-blood lymphocyte cytotoxicity was seen in 0/6 (0%) Dukes' A, 5/22 (23%) Dukes' B and 9/32 (28%) Dukes' C cases. Thus, whilst migration of specifically cytotoxic E-rosetting lymphocytes from the bloodstream to the tumour may occur eventually, it may equally be true that stromal intrinsic immunoreactivity is a relatively early event in tumour development. It could theoretically result from initial infiltration and cellular trapping of unprimed nonspecific inflammatory cells, possibly with specific cytotoxic T cells from the blood accompanied by intrinsic lymphocyte activation and proliferation.

The correlation of intrinsic (*i.e.* stromal) E-rosetting lymphocyte anti-tumour reactivity with the presence of perivascular cuffing by lymphocytes suggests that immunoreactive cells may first become localized mainly at the tumour edge, although these perivascular cells have not yet been characterized immunologically. It may also be relevant that the presence of such perivascular cuffs of small lymphocytes is associated with prolonged recurrence-free survival in Stage B colorectal carcinoma (Pihl *et al.*, 1977). Consequently both intrinsic E-rosetting lymphocyte cytotoxicity and stromal perivascular cuffing may reasonably be assumed to reflect beneficial local immunocyte anti-tumour immunoreactivity.

Much of this work formed part of studies for Ph.D. candidature by J. A. Werkmeister. The work was supported by the Anti-Cancer Council of Victoria. We thank the surgeons, Mr A. M. Cuthbertson, Sir Edward Hughes and Mr A. J. Rollo for providing the tumour specimens. We also thank Mrs L. Campbell and Mrs W. Shepherd-Clark for technical assistance.

APPENDIX I.—*Effect of E-rosetting fractionation on* in vitro *natural non-specific lymphocyte reactivity against tumour cells*

| Exp. | Lymphocyte fraction | Mean tumour cells per well ± s.d. |
|---|---|---|
| 1 | E-rosetted | 20·5 ± 3·7 |
| | Non E-rosetting | 22·3 ± 5·7 |
| | Unfractionated | 21·3 ± 4·1 |
| | No lymphocytes | 22·9 ± 4·6 |
| 2 | E-rosetted | 16·7 ± 6·7 |
| | Non E-rosetting | 15·2 ± 4·6 |
| | Unfractionated | 17·3 ± 5·7 |
| | No lymphocytes | 18·0 ± 3·1 |
| 3 | E-rosetted | 28·0 ± 5·0 |
| | Non E-rosetting | 25·8 ± 6·0 |
| | Unfractionated | 27·2 ± 3·6 |
| | No lymphocytes | 28·1 ± 6·2 |
| 4 | E-rosetted | 34·8 ± 5·8 |
| | Non E-rosetting | 35·7 ± 7·0 |
| | Unfractionated | 36·8 ± 2·9 |
| | No lymphocytes | 33·2 ± 4·9 |
| 5 | E-rosetted | 32·8 ± 4·7 |
| | Non E-rosetting | 36·2 ± 8·5 |
| | Unfractionated | 33·2 ± 4·0 |
| | No lymphocytes | 30·9 ± 4·0 |

Effector : target cell ratio, 100:1.

APPENDIX II.—*Effect of E-rosette fractionation on phytohaemagglutinin (PHA) stimulation of normal lymphocytes*

| | | $^3$H-thymidine uptake (ct/min × $10^{-3}$) | |
|---|---|---|---|
| Exp. | Lymphocyte fraction | Without PHA | With PHA |
| 1 | Unfractionated | 1·6 | 135·5 |
| | E-rosetted | 0·8 | 49·0** |
| | Non E-rosetting | 2·4 | 5·7*** |
| | E and non-E re-constituted | 1·5 | 122·5 |
| 2 | Unfractionated | 1·1 | 83·5 |
| | E-rosetted | 0·9 | 25·6** |
| | Non E-rosetting | 2·2* | 5·0*** |
| | E and non-E re-constituted | 1·2 | 78·6 |
| 3 | Unfractionated | 1·0 | 68·5 |
| | E-rosetted | 1·0 | 21·4** |
| | E and non-E re-constituted | 2·0 | 59·6 |

Significance of difference from unfractionated cells: * $P < 0·05$; ** $P < 0·01$; *** $P < 0·001$.

APPENDIX III.—*Protocol of intrinsic lymphocyte cytotoxicity testing*

| | Mean no. tumour cells ± s.d. | % Cytotoxicity |
|---|---|---|
| Patient A. (54% E, 40% EAC) | | |
| Blank | 30·8 ± 5·1 | |
| Normal lymphocytes | 27·5 ± 6·6 | — |
| E-rosetted intrinsic lymphocytes | 16·7 ± 2·7 | 39·4 |
| EAC-rosetted intrinsic lymphocytes | 28·3 ± 7·7 | — |
| Patient B. (70% E, 15% EAC) | | |
| Blank | 38·5 ± 4·4 | |
| Normal lymphocytes | 36·8 ± 2·9 | — |
| E-rosetted intrinsic lymphocytes | 35·7 ± 5·9 | 3·2 |
| EAC-rosetted intrinsic lymphocytes | 36·1 ± 8·5 | 1·8 |
| Patient C. (48% E, 32% EAC) | | |
| Blank | 39·5 ± 3·4 | |
| Normal lymphocytes | 40·0 ± 1·3 | — |
| E-rosetted intrinsic lymphocytes | 23·7 ± 2·8 | 40·8 |
| EAC-rosetted intrinsic lymphocytes | 41·8 ± 6·4 | −4·6 |

### REFERENCES

VAN BOXEL, J. A., PAUL, W. E., FRANK, M. M. & GREEN, I. (1973) Antibody-dependent lymphoid cell-mediated cytotoxicity: Role of lymphocytes bearing a receptor for complement. *J. Immunol.*, **110**, 1027.

CHALKLEY, H. W. (1943) Method for the quantitative morphologic analysis of tissues. *J. Natl Cancer Inst.*, **4**, 47.

CURRIE, G. A. & BASHAM, C. (1972) Serum mediated inhibition of the immunological reactions of the patient to his own tumour: A possible role for circulating antigen. *Br. J. Cancer*, **26**, 427.

DUKES, C. E. (1960) Cancer of the rectum. In *Neoplastic Disease at Various Sites: III*. Ed. Smither & Dukes. Edinburgh: Livingstone. p. 59.

JONDAL, M., HOLM, G. & WIGZELL, H. (1972) Surface markers on human B and T lymphocytes. A large population of lymphocytes forming non-immune rosettes with sheep red blood cells. *J. Exp. Med.*, **136**, 207.

JONDAL, M., SVEDMYR, E., KLEIN, E. & SINGH, S. (1975) Killer T cells in a Burkitt's lymphoma biopsy. *Nature*, **255**, 405.

MANNICK, J. A., CONSTANTIAN, M., PARDRIDGE, E., SAPOROSCHETZ, I. & BADGER, A. (1977) Improvement of phytohaemagglutinin responsiveness of lymphocytes from cancer patients after washing *in vitro*. *Cancer Res.*, **37**, 3066.

MATTHEWS, N. & MACLAURIN, B. P. (1973) Non-thymic origin of lymphocytes mediating antibody-induced cytotoxicity against tumor cells. *Proc. Univ. Otago Med. Sch.*, **51**, 13.

MATTHEWS, N., ROLLAND, J. M. & NAIRN, R. C. (1976) Lymphoid cell fractionation by aggregated immunoglobulin-agarose column. *J. Immunol. Meth.*, **9**, 323.

NAIRN, R. C. (1976) Immunological Reactions in Human Cancer (3): Carcinoma of colon, and squamous cell carcinoma of skin. In *Scientific Foundations of Oncology*. Eds. Symington & Carter. London: Heinemann. p. 549.

NAIRN, R. C., NIND, A. P. P., GULI, E. P. G. & 4 others (1971) Immunological reactivity in patients with carcinoma of colon. *Br. Med. J.*, iv, 706.

NIND, A. P. P., MATTHEWS, N., PIHL, E. A. V., ROLLAND, J. M. & NAIRN, R. C. (1975) Analysis of inhibition of lymphocyte cytotoxicity in human colon carcinoma. *Br. J. Cancer*, **31**, 620.

NIND, A. P. P., NAIRN, R. C., ROLLAND, J. M., GULI, E. P. G. & HUGHES, E. S. R. (1973) Lymphocyte anergy in patients with carcinoma. *Br. J. Cancer*, **28**, 108.

PIHL, E., MALAHY, M. A., KHANKHANIAN, N., HERSH, E. M. & MAVLIGIT, G. M. (1977) Immunomorphological features of prognostic significance in Dukes' class B colorectal carcinoma. *Cancer Res.*, **37**, 4145.

POTTER, M. R. & MOORE, M. (1979) Natural cytotoxic reactivity of human lymphocyte subpopulations. *Immunology*, **37**, 187.

UNDERWOOD, J. C. E. (1974) Lymphoreticular cell infiltration in human tumours prognostic and biological implications: A review. *Br. J. Cancer*, **30**, 538.

VOSE, B. M., VANKY, F. & KLEIN, E. (1977) Human tumor–lymphocyte interaction *in vitro*. V. Comparison of the reactivity of tumor-infiltrating, blood and lymph-node lymphocytes with autologous tumor cells. *Int. J. Cancer*, **20**, 895.

WEIBEL, E. R. (1963) Principles and methods for the morphometric study of the lung and other organs. *Lab. Invest.*, **12**, 131.